# Cardiovascular disease risk in adults with compromised bone health

**Published:** 2022-10

**SEPTEMBER 08, 2022** - In a study published in the *Journal of Bone and Mineral Research*, investigators identified several factors that may put adults with compromised bone health - such as individuals with osteoporosis - at risk for experiencing heart attacks and strokes.

These risk factors included male gender, older age, smoking, alcohol consumption, atrial fibrillation, use of anti-hypertensive medications, prior heart attack or stroke, established cardiovascular disease, low kidney function, high systolic blood pressure, elevated cholesterol level, and use of multiple concomitant medicines.

“Although there are some calculators to produce risk estimates of cardiovascular disease, these are not targeted at those at high risk of fracture,” said corresponding author Daniel Prieto-Alhambra, MD, PhD, of the University of Oxford, in the UK. “To our knowledge, this is the first study to identify cardiovascular disease risk factors for osteoporotic individuals using data that is routinely collected and readily available.”

*Full Citation: “Estimating the incidence and key risk factors of cardiovascular disease in patients at high risk of imminent fracture using routinely collected real-world data From the UK” Marta Pineda-Moncusí, Leena El-Hussein, Antonella Delmestri, Cyrus Cooper, Alireza Moayyeri, Cesar Libanati, Emese Toth, Daniel Prieto-Alhambra, Sara Khalid. JBMR; Published Online: July 11, 2022 (DOI: 10.1002/jbmr.4648).*

*URL Upon Publication: https://onlinelibrary.wiley.com/doi/10.1002/jbmr.4648*

*Copyright © 2021 The Cochrane Collaboration. Published by John Wiley & Sons, Ltd., reproduced with permission.*

